# Red Blood Cell Transfusion in European Neonatal Intensive Care Units, 2022 to 2023

**DOI:** 10.1001/jamanetworkopen.2024.34077

**Published:** 2024-09-19

**Authors:** Nina A. M. Houben, Suzanne Fustolo-Gunnink, Karin Fijnvandraat, Camila Caram-Deelder, Marta Aguar Carrascosa, Alain Beuchée, Kristin Brække, Francesco Cardona, Anne Debeer, Sara Domingues, Stefano Ghirardello, Ruza Grizelj, Emina Hadžimuratović, Christian Heiring, Jana Lozar Krivec, Jan Malý, Katarina Matasova, Carmel Maria Moore, Tobias Muehlbacher, Miklos Szabó, Tomasz Szczapa, Gabriela Zaharie, Justine de Jager, Nora Johanna Reibel-Georgi, Helen V. New, Simon J. Stanworth, Emöke Deschmann, Charles C. Roehr, Christof Dame, Saskia le Cessie, Johanna van der Bom, Enrico Lopriore

**Affiliations:** 1 Sanquin Research, Sanquin Blood Supply Foundation, Amsterdam, the Netherlands; 2 Division of Neonatology, Willem-Alexander Children’s Hospital, Leiden University Medical Center, Leiden, the Netherlands; 3 Pediatric Hematology, Emma Children’s Hospital, Amsterdam University Medical Center, University of Amsterdam, Amsterdam, the Netherlands; 4 Sanquin Research, Department of Molecular Cellular Hemostasis, Amsterdam, the Netherlands; 5 Department of Clinical Epidemiology, Leiden University Medical Center, Leiden, the Netherlands; 6 Department of Neonatology, La Fe University Hospital, Valencia, Spain; 7 Department of Pediatrics, Centre Hospitalier Universitaire de Rennes, Rennes, France; 8 Women and Children's Division, Department of Neonatal Intensive Care, Oslo University Hospital, Oslo, Norway; 9 Division of Neonatology, Medical University Vienna, Vienna, Austria; 10 Department of Neonatology, Universitair Ziekenhuis Leuven, Leuven, Belgium; 11 Centro Materno-Infantil do Norte–Unidade Local de Saúde de Santo António, Porto, Portugal; 12 Fondazione Istituto di Ricovero e Cura a Carattere Scientifico, Policlinico San Matteo, Pavia, Italy; 13 Department of Pediatrics, University Hospital Centre Zagreb, School of Medicine, University of Zagreb, Zagreb, Croatia; 14 Paediatric Clinic, University Medical Center Sarajevo, Sarajevo, Bosnia and Herzegovina; 15 Department of Neonatal and Paediatric Intensive Care, Copenhagen University Hospital, Rigshospitalet, Denmark; 16 Faculty of Medicine, University of Ljubljana, Ljubljana, Slovenia; 17 Department of Neonatology, University Children’s Hospital, University Medical Centre Ljubljana, Ljubljana, Slovenia; 18 Department of Pediatrics, University Hospital Hradec Králové, Hradec Králové, Czech Republic; 19 Jessenius Faculty of Medicine, University Hospital Martin, Martin, Slovakia; 20 School of Medicine, University College Dublin, Dublin, Ireland; 21 National Maternity Hospital, Dublin, Ireland; 22 Department of Neonatology, University Hospital Zurich, Zurich, Switzerland; 23 Department of Neonatology, Semmelweis University, Budapest, Hungary; 24 II Department of Neonatology, Poznan University of Medical Sciences, Poznan, Poland; 25 University of Medicine and Pharmacy Iuliu Hatieganu, Cluj-Napoca, Romania; 26 Charité–Universitätsmedizin Berlin, Berlin, Germany; 27 NHS (National Health Service) Blood and Transplant, London, United Kingdom; 28 Radcliffe Department of Medicine, University of Oxford, Oxford, United Kingdom; 29 Department of Neonatology, Karolinska Institute, Stockholm, Sweden; 30 National Perinatal Epidemiology Unit, Oxford Population Health, University of Oxford, Oxford, United Kingdom; 31 Faculty of Health Sciences, University of Bristol, Bristol, United Kingdom; 32 Women’s and Children’s Division, Southmead Hospital, North Bristol NHS Trust, Bristol, United Kingdom

## Abstract

**Importance:**

Red blood cell (RBC) transfusions are frequently administered to preterm infants born before 32 weeks of gestation in the neonatal intensive care unit (NICU). Two randomized clinical trials (Effects of Transfusion Thresholds on Neurocognitive Outcomes of Extremely Low-Birth-Weight Infants [ETTNO] and Transfusion of Prematures [TOP]) found that liberal RBC transfusion thresholds are nonsuperior to restrictive thresholds, but the extent to which these results have been integrated into clinical practice since publication in 2020 is unknown.

**Objective:**

To describe neonatal RBC transfusion practice in Europe.

**Design, Setting, and Participants:**

This international prospective observational cohort study collected data between September 1, 2022, and August 31, 2023, with a 6-week observation period per center, from 64 NICUs in 22 European countries. Participants included 1143 preterm infants born before 32 weeks of gestation.

**Exposure:**

Admission to the NICU.

**Main Outcomes and Measures:**

Study outcome measures included RBC transfusion prevalence rates, cumulative incidence, indications, pretransfusion hemoglobin (Hb) levels, volumes, and transfusion rates, Hb increment, and adverse effects of RBC transfusion.

**Results:**

A total of 1143 preterm infants were included (641 male [56.1%]; median gestational age at birth, 28 weeks plus 2 days [IQR, 26 weeks plus 2 days to 30 weeks plus 2 days]; median birth weight, 1030 [IQR, 780-1350] g), of whom 396 received 1 or more RBC transfusions, totaling 903 transfusions. Overall RBC transfusion prevalence rate during postnatal days 1 to 28 was 3.4 transfusion days per 100 admission days, with considerable variation across countries, only partly explained by patient mix. By day 28, 36.5% (95% CI, 31.6%-41.5%) of infants had received at least 1 transfusion. Most transfusions were given based on a defined Hb threshold (748 [82.8%]). Hemoglobin levels before transfusions indicated for threshold were below the restrictive thresholds set by ETTNO in 324 of 729 transfusions (44.4%) and TOP in 265 of 729 (36.4%). Conversely, they were between restrictive and liberal thresholds in 352 (48.3%) and 409 (56.1%) transfusions, respectively, and above liberal thresholds in 53 (7.3%) and 55 (7.5%) transfusions, respectively. Most transfusions given based on threshold had volumes of 15 mL/kg (470 of 738 [63.7%]) and were administered over 3 hours (400 of 738 [54.2%]), but there was substantial variation in dose and duration.

**Conclusions and Relevance:**

In this cohort study of very preterm infants, most transfusions indicated for threshold were given for pretransfusion Hb levels above restrictive transfusion thresholds evaluated in recent trials. These results underline the need to optimize practices and for implementation research to support uptake of evidence.

## Key Points

**Question:**

What is the current red blood cell (RBC) transfusion practice for preterm infants born before 32 weeks’ gestation in Europe?

**Findings:**

This cohort study included 1143 infants from 64 neonatal intensive care units across 22 European countries. By day 28 of life, 36.5% of infants had received an RBC transfusion, and most transfusions based on hemoglobin threshold were given above restrictive thresholds tested in recent trials.

**Meaning:**

These findings suggest that there is a need to address the gap between evidence and practice and to understand factors influencing ongoing variable practices of RBC transfusions among preterm infants.

## Teaser

This cohort study describes rates, indications, volumes, increments, and adverse effects of red blood cell transfusion for preterm infants born before 32 weeks' gestation in European neonatal intensive care units.

## Introduction

Red blood cell (RBC) transfusions are often administered to very preterm infants (gestational age at birth, <32 weeks) in the neonatal intensive care unit (NICU). Two large randomized clinical trials (RCTs) published in 2020—the Effects of Transfusion Thresholds on Neurocognitive Outcomes of Extremely Low-Birth-Weight Infants (ETTNO) trial^1^ and the Transfusion of Prematures (TOP) trial^2^—compared liberal vs restrictive RBC transfusion thresholds. Both trials concluded that liberal thresholds were not superior to restrictive thresholds in terms of mortality or neurodevelopmental impairment at 2 years of corrected age. Additionally, a post hoc analysis of the Preterm Erythropoietin Neuroprotection Trial^3^ also suggested possibly deleterious effects of RBC transfusions on neurodevelopmental outcomes of preterm infants.

An earlier survey by the Neonatal Transfusion Network^4^ conducted during and shortly after publication of the RCTs found evidence of significant variation in transfusion practices across European NICUs. However, the timing of this survey did not allow assessment of the implementation of more restrictive RBC transfusion thresholds based on recent trial data. Epidemiological, patient-level data are an essential step in understanding how hemoglobin (Hb) threshold evidence translates into practice and the variability of neonatal transfusion practices.^5,6,7^ Considerable uncertainty also applies for other aspects of transfusion management in preterm infants such as transfusion volumes. Therefore, we conducted a prospective observational study across 64 NICUs in 22 countries to describe RBC transfusion rates, indications, volumes, increments, and adverse effects in Europe and to evaluate clinical Hb transfusion triggers compared with recent RCTs.

## Methods

### Design

We performed a prospective, observational cohort study (International Neonatal Transfusion Point Prevalence [INSPIRE]) to assess transfusion practice in multiple European NICUs. The Medical Research Ethics Committee of the Leiden University Medical Center, Leiden, the Netherlands, approved the study, followed by national or regional ethics boards in the participating countries. Study conduct complied with the Declaration of Helsinki^8^ and the General Data Protection Regulation.^9^ We established a parental advisory board in collaboration with the European Foundation for the Care of the Newborn Infant. The study protocol and statistical analysis plan are available in the ISRCTN registry (registration number ISRCTN17267090). Parents and guardians provided informed consent (oral or written) if required by regional or national legislation. This study followed the Reporting of Observational Studies in Epidemiology (STROBE) reporting guidelines.

### Data Collection

Data were obtained between September 1, 2022, and August 31, 2023; in all centers data were collected during a 6-week study period. We included all infants born at a gestational age younger than 32 weeks and admitted to the NICU during the study period. Infants were enrolled at the latest of the following times: NICU admission, start of study period, or date of consent. Infants were followed up until death, discharge, or end of study period, whichever occurred first. An RBC transfusion day was defined as any admission day in the NICU during the study period on which the infant received at least 1 RBC transfusion. We collected the following information for each transfusion: primary indication, volume and duration, ventilation status during transfusion, and Hb or hematocrit (Hct) levels closest before and after transfusion (within 24 hours from transfusion). Transfusion-associated adverse effects potentially linked to the preceding transfusion could be registered in a free text format. Additionally, we recorded any event of major bleeding, culture-confirmed sepsis, necrotizing enterocolitis, invasive mechanical ventilation, and surgery occurring during the study period (definitions are given in eTable 1 in Supplement 1).

### Outcomes

Study outcome measures included (1) observed and expected RBC transfusion day prevalence rates, separately for during and after the first 28 postnatal days of life; (2) cumulative incidence of receiving at least 1 RBC transfusion during the first 28 postnatal days of life, adjusted for the competing risks of death and discharge; (3) primary indications for transfusion; (4) volume, duration, and infusion rate of transfusion; (5) Hb levels prior to transfusion; (6) transfusion Hb increment; and (7) transfusion-related adverse effects. RBC transfusion day prevalence rate was defined as the number of RBC transfusion days per 100 NICU admission days. This definition accounted for the different length of follow-up between infants inherent to the dynamic cohort study design. Expected RBC transfusion day prevalence rates based on patient mix were presented to facilitate comparison of prevalence rates between countries. All data were collected using a certified electronic database (Castor EDC; Castor) that complies with International Council for Harmonisation of Technical Requirements for Pharmaceuticals for Human Use, E6 Good Clinical Practice standards.^10^

### Statistical Analysis

Patient and hospital characteristics were presented as mean (SD), median (IQR), or frequency (percentage). For the observed prevalence rate, we performed a meta-analysis (using the function metarate from the R package meta; R, version 4.1.17 [R Project for Statistical Computing]) to pool RBC transfusion day prevalence rates from the individual centers into subgroup estimates per country and subsequently to derive the overall estimate. Given that we expected large variability between countries, we used random-effects Poisson models as described by Stijnen and colleagues^11^ to account for this heterogeneity.

Expected prevalence rates adjusted for patient mix per country were calculated using logistic regression with transfusion day as dependent variable and several independent variables, including sex, multiple gestation, gestational age at birth, birth weight, postnatal day, major congenital anomalies, bleeding disorders, major bleeding, necrotizing enterocolitis, sepsis, mechanical ventilation, and surgery. This model was used to estimate the probability of receiving at least 1 RBC transfusion per infant per day, and these estimates were then averaged to obtain the expected prevalence rate per country.

We computed the cumulative incidence of receiving at least 1 RBC transfusion during the first 28 days of life (nonparametric Aalen-Johansen estimate, using function cuminc from the package cmprsk [R, version 4.1.17]), while considering death and discharge as competing events.^12^ In this analysis, only infants who were followed up from birth were included.

Infusion rate (in milliliters per kilogram per hour) was calculated by dividing the transfusion volume (in milliliters per kilogram) by transfusion duration (in hours). All Hb and Hct levels were converted to Hb in grams per liter using the following conversions: Hb (g/L) = Hb (mmol/L) × 16, and Hb (g/L) = Hct (%)/0.3. We compared the pretransfusion Hb values with the liberal and restrictive thresholds as evaluated in the 2 most recent large RCTs (thresholds are available in eTable 2 in Supplement 1).^1,2^ Transfusion increment was calculated by subtracting posttransfusion Hb levels from pretransfusion Hb levels (all within 24 hours before or after transfusion).

Statistical analyses for the main outcome measures were conducted in R statistical software, version 4.1.17. Computations for the additional study outcome measures were performed using Stata statistical Software, version 16.1 (StataCorp LLC). Figures were created using Prism, version 9.3.1 (GraphPad).

## Results

### Patients and Centers

A total of 1143 patients (502 [43.9%] female and 641 [56.1%] male) from 64 centers in 22 European countries were included, with a median gestational age at birth of 28 weeks plus 2 days (IQR, 26 weeks plus 2 days to 30 weeks plus 2 days) and median birth weight of 1030 (IQR, 780-1350) g. A median of 17 (IQR, 11-25) patients per center were included, with a median study follow-up per patient of 20 (IQR, 10-35) days. Seventy-two patients (6.3%) died during study follow-up (median postnatal age at death, 8 [IQR, 4-18] days). Characteristics of the study population are shown in the Table, and characteristics of the participating centers are shown in eTable 3 in Supplement 1.

**Table.** Patient Characteristics

Country	No. (% of total)		Median (IQR)				No./total No. (%) of patients								
	Patients	Total follow-up, d	Follow-up per patient, d	Postnatal age at inclusion, d	Gestational age at birth, wk plus d	Birth weight, g	Female sex	Multifetal pregnancy	Congenital anomaly	Bleeding disorder	Major bleeding^a^	NEC^a^	Sepsis^a^	Invasive mechanical ventilation^b^	Surgery^c^
Austria	50 (4.4)	1397 (5.6)	29 (14-42)	13 (1-36)	26 + 4 (24 + 4 to 28 + 5)	890 (650-1280)	22/50 (44.0)	15/50 (30.0)	2/50 (4.0)	0/50	5/50 (10.0)	0/50	7/50 (14.0)	12/50 (24.0)	5/50 (10.0)
Belgium	16 (1.4)	283 (1.1)	13 (6-32)	8 (43-80)	29 + 6 (25 + 4 to 30 + 6)	1180 (852-1357)	9/16 (56.3)	9/16 (56.3)	0/16	0/16	0/16	0/16	0/16	1/16 (6.3)	1/16 (6.3)
Bosnia and Herzegovina	32 (2.8)	648 (2.6)	22 (10-31)	1 (1-21)	29 + 6 (28 + 2 to 31 + 1)	1252 (1015-1592)	17/32 (53.1)	8/32 (25.0)	2/32 (6.3)	0/32	3/32 (9.4)	0/32	3/32 (9.4)	6/32 (18.8)	1/32 (3.1)
Croatia	24 (2.1)	647 (2.6)	32 (18-34)	1 (1-23)	30 + 3 (28 + 1 to 31 + 4)	1225 (1075-1440)	17/24 (70.8)	10/24 (41.7)	0/24	0/24	1/24 (4.2)	0/24	2/24 (8.3)	3/24 (12.5)	1/24 (4.2)
Czech Republic	53 (4.6)	1173 (4.7)	18 (13-32)	5 (1-30)	30 + 1 (27 + 5 to 31 + 2)	1230 (925-1600)	21/53 (39.6)	17/53 (32.1)	2/53 (3.8)	0/53	1/53 (1.9)	0/53	3/53 (5.7)	10/53 (18.9)	3/53 (5.7)
Denmark	34 (3.0)	615 (2.5)	14 (5-24)	3 (1-44)	27 + 0 (25 + 5 to 29 + 0)	940 (788-1200)	14/34 (41.2)	12/34 (35.3)	3/34 (8.8)	0/34	3/34 (8.8)	1/34 (2.9)	6/34 (17.6)	18/34 (52.9)	7/34 (20.6)
France	116 (10.1)	2664 (10.7)	21 (11-37)	4 (1-32)	28 + 4 (26 + 2 to 30 + 2)	1047 (780-1335)	50/116 (43.1)	26/116 (22.4)	3/116 (2.6)	0/116	10/116 (8.6)	4/116 (3.4)	19/116 (16.4)	40/116 (34.5)	9/116 (7.8)
Germany	76 (6.6)	1951 (7.8)	25 (13-42)	17 (1-62)	26 + 4 (25 + 2 to 29 + 4)	845 (605-1190)	33/76 (43.4)	24/76 (31.6)	5/76 (6.6)	0/76	2/76 (2.6)	0/76	5/76 (6.6)	20/76 (26.3)	6/76 (7.9)
Hungary	39 (3.4)	757 (3.0)	18 (13-27)	3 (1-18)	27 + 2 (26 + 1 to 30 + 2)	920 (750-1160)	18/39 (46.2)	8/39 (20.5)	1/39 (2.6)	0/39	6/39 (15.4)	3/39 (7.7)	13/39 (33.3)	13/39 (33.3)	2/39 (5.1)
Ireland	61 (5.3)	1178 (4.7)	16 (6-32)	1 (1-13)	26 + 4 (25 + 2 to 29 + 4)	1090 (850-1400)	29/61 (47.5)	18/61 (29.5)	1/61 (1.6)	0/61	5/61 (8.2)	6/61 (9.8)	7/61 (11.5)	31/61 (50.8)	5/61 (8.2)
Italy	124 (10.8)	3178 (12.7)	27 (12-41)	21 (1-51)	28 + 4 (26 + 4 to 30 + 0)	1050 (817-1290)	46/124 (37.1)	39/124 (31.5)	3/124 (2.4)	3/124 (2.4)	11/124 (8.9)	3/124 (2.4)	9/124 (7.3)	36/124 (29.0)	13/124 (10.5)
The Netherlands	79 (6.9)	1280 (5.1)	10 (6-24)	1 (1-10)	28 + 4 (26 + 3 to 30 + 4)	1145 (886-1500)	32/79 (40.5)	23/79 (29.1)	2/79 (2.5)	0/79	4/79 (5.1)	0/79	8/79 (10.1)	25/79 (31.6)	4/79 (5.1)
Norway	33 (2.9)	567 (2.3)	12 (5-32)	5 (1-31)	26 + 0 (25 + 1 to 26 + 6)	793 (636-1010)	15/33 (45.5)	12/33 (36.4)	0/33	1/33 (3.0)	8/33 (24.2)	1/33 (3.0)	5/33 (15.2)	18/33 (54.5)	4/33 (12.1)
Poland	84 (7.3)	1969 (7.9)	27 (9-37)	20 (1-41)	28 + 1 (26 + 6 to 30 + 1)	1085 (850-1345)	33/84 (39.3)	24/84 (28.6)	3/84 (3.6)	0/84	10/84 (11.9)	3/84 (3.6)	12/84 (14.3)	36/84 (42.9)	14/84 (16.7)
Portugal	19 (1.7)	364 (1.5)	19 (5-29)	15 (3-49)	29 + 5 (28 + 6 to 31 + 1)	990 (810-1410)	12/19 (63.2)	4/19 (21.1)	0/19	0/19	0/19	0/19	2/19 (10.5)	4/19 (21.1)	0/19
Romania	29 (2.5)	688 (2.8)	23 (14-33)	4 (1-12)	29 + 0 (27 + 4 to 31 + 0)	1300 (880-1600)	1/29 (3.4)	11/29 (37.9)	3/29 (10.3)	0/29	7/29 (24.1)	1/27 (3.7)	4/29 (13.8)	17/29 (58.6)	0/29
Slovakia	30 (2.6)	783 (3.1)	27 (12-39)	7 (3-38)	27 + 6 (25 + 6 to 30 + 2)	940 (780-1280)	17/30 (56.7)	4/30 (13.3)	2/30 (6.7)	0/30	0/30	1/30 (3.3)	3/30 (10.0)	9/30 (30.0)	1/30 (3.3)
Slovenia	20 (1.7)	359 (1.4)	15 (7-26)	23 (1-55)	28 + 3 (26 + 4 to 29 + 6)	895 (730-1347)	10/20 (50.0)	6/20 (30.0)	1/20 (5.0)	0/20	1/20 (5.0)	0/20	0/20	6/20 (30.0)	1/20 (1.0)
Spain	87 (7.6)	1771 (7.1)	17 (10-31)	1 (1-23)	28 + 5 (26 + 4 to 30 + 2)	1000 (770-1285)	36/87 (41.4)	28/87 (32.2)	1/87 (1.1)	0/87	5/87 (5.7)	2/87 (2.3)	9/87 (10.3)	16/87 (18.4)	6/87 (6.9)
Sweden	49 (4.3)	830 (3.3)	15 (5-26)	4 (1-11)	28 + 3 (25 + 5 to 29 + 6)	1060 (745-1396)	20/49 (40.8)	16/49 (32.7)	4/49 (8.2)	0/49	3/49 (6.1)	5/49 (10.2)	2/49 (4.1)	22/49 (44.9)	6/49 (12.2)
Switzerland	50 (4.4)	1010 (4.0)	17 (8-33)	2 (1-18)	28 + 4 (26 + 3 to 30 + 4)	1120 (800-1470)	24/50 (48.0)	14/50 (28.0)	3/50 (6.0)	2/50 (4.0)	6/50 (12.0)	1/50 (2.0)	2/50 (4.0)	19/50 (38.0)	2/50 (4.0)
United Kingdom	38 (3.3)	866 (3.5)	23 (12-36)	3 (1-20)	26 + 6 (24 + 3 to 29 + 2)	883 (618-1120)	12/38 (31.6)	8/38 (21.1)	2/38 (5.3)	0/38	1/38 (2.6)	2/38 (5.3)	4/38 (10.5)	21/38 (55.3)	7/38 (18.4)
Overall	1143 (100)	24 978 (100)	20 (10-35)	5 (1-31)	28 + 2 (26 + 2 to 30 + 2)	1030 (780-1350)	502/1143 (43.9)	336/1143 (29.4)	43/1143 (3.8)	6/1143 (0.5)	92/1143 (8.0)	33/1143 (2.9)	125/1143 (10.9)	383/1143 (33.5)	98/1143 (8.6)

Abbreviation: NEC, necrotizing enterocolitis.

^a^ Indicates at least 1 episode during study follow-up. See eTable 1 in Supplement 1 for definition.

^b^ Indicates at least 1 day during study follow-up. See eTable 1 in Supplement 1 for definition.

^c^ Indicates any type during study follow-up. See eTable 1 in Supplement 1 for definition.

### Transfusions

**Figure 1.**
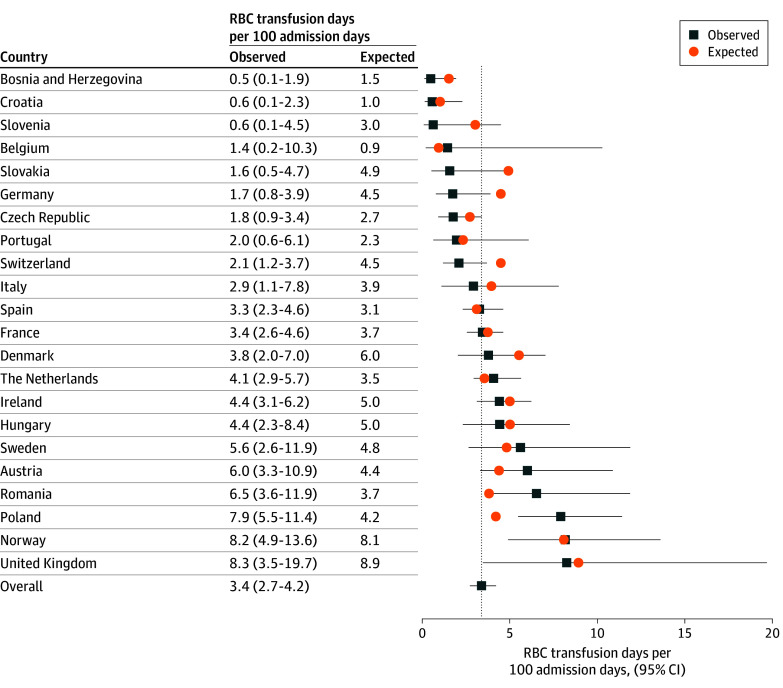
Red Blood Cell (RBC) Transfusion Day Prevalence Rates During the First 28 Postnatal Days Observed prevalence rates were calculated using random-effects Poisson models to pool RBC transfusion day prevalence rates from the individual centers into country subgroup estimates and subsequently to derive the overall estimate (represented by the dotted vertical line) with 95% CIs). Expected RBC transfusion day prevalence rates as estimated were based on patient mix using a logistic regression model that included the following variables: sex, multiple gestation, gestational age at birth, birth weight, postnatal day, major congenital anomalies, bleeding disorders, major bleeding, necrotizing enterocolitis, sepsis, mechanical ventilation, and surgery.

Among the 1143 patients, 396 (34.6%) received 1 or more RBC transfusions during the study; in total, 903 transfusions were given. The median number of transfusions per infant was 2.0 (IQR, 1.0-3.0). The overall observed RBC transfusion prevalence rate during first 28 days of life was 3.4 (95% CI, 2.7-4.2) RBC transfusion days per 100 admission days (Figure 1), and 2.0 (95% CI, 1.6-2.6) RBC transfusion days per 100 admission days after the first 28 days of life (eFigure 1 in Supplement 1). Observed and expected prevalence rates per country during days 1 to 28 and after day 28 are also highlighted in Figure 1 and eFigure 1 in Supplement 1, respectively. Regression coefficients for estimating expected prevalence rates are provided in eTable 4 in Supplement 1.

**Figure 2.**
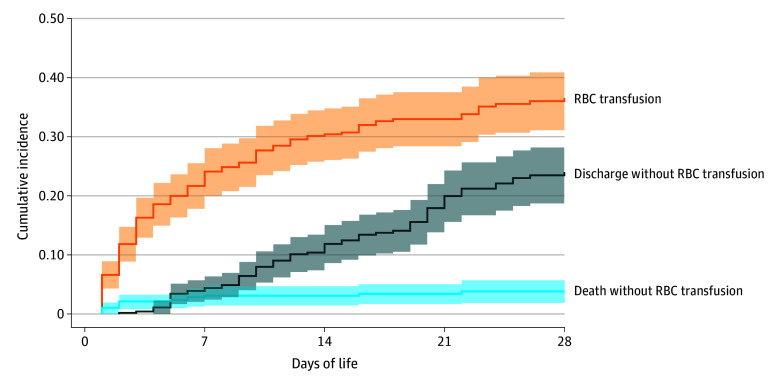
Cumulative Incidence of Receiving at Least 1 Red Blood Cell (RBC) Transfusion During First 28 Days of Life Adjusted for the competing risks of death and discharge (with corresponding 95% CIs, indicated by shaded areas), based on 468 of 1143 infants (40.9%) who were followed up from birth.

The proportion of infants who had received at least 1 RBC transfusion by day 7 of life was 24.1% (95% CI, 20.1%-28.1%); by day 28, 36.5% (95% CI, 31.6%-41.5%). Death and discharge were considered as competing events. Proportions were based on 468 of 1143 infants (40.9%) followed up from birth (Figure 2).

### Transfusion Indication

**Figure 3.**
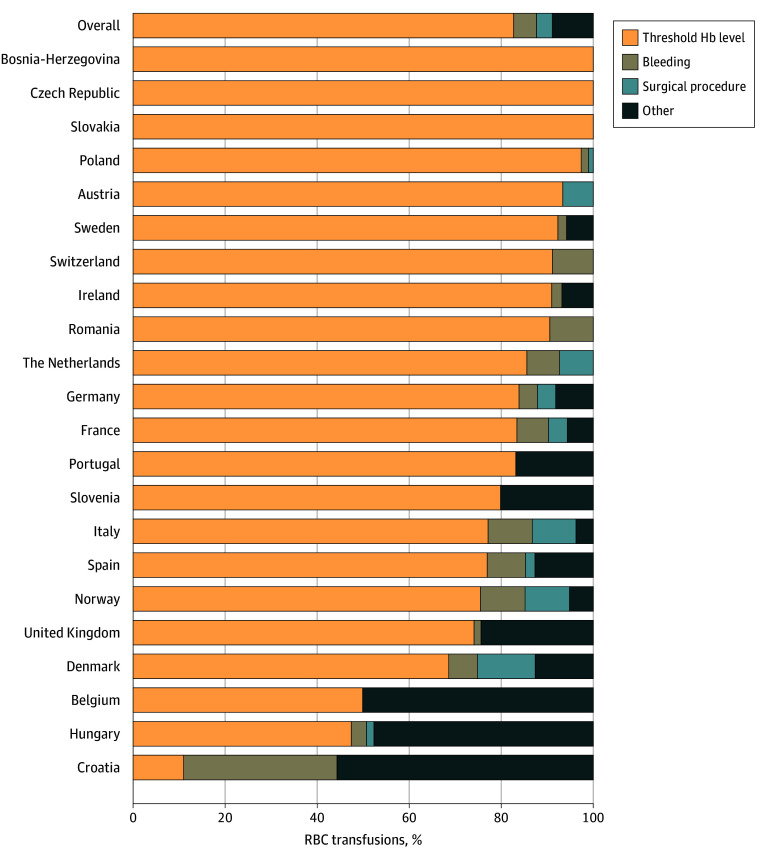
Primary Indications for 903 Red Blood Cell (RBC) Transfusions Other indications included critically ill conditions, increased respiratory support, apnea, hypotension, cardiac arrest, blood sampling threshold, patent ductus arteriosus, inadequate weight gain, tachypnea, and ventricular septal defect. Hb indicates hemoglobin.

As shown in Figure 3, the most common primary indication for RBC transfusion was Hb threshold (748 of 903 [82.8%]). For a smaller proportion of transfusions, the primary indications were active bleeding (45 of 903 [5.0%]), surgical procedures (31 of 903 [3.4%]), and other (79 of 903 [8.8%]). The other primary indications were critically ill conditions (26 of 903 [2.9%]), increased respiratory support (15 of 903 [1.7%]), apnea (9 of 903 [1.0%]), hypotension (9 of 903 [1.0%]), cardiac arrest (7 of 903 [0.8%]), compensation for blood loss for laboratory or diagnostic tests (based on sample blood volume, irrespective of Hb level) (5 of 903 [0.6%]), patent ductus arteriosus (5 of 903 [0.6%]), inadequate weight gain (1 of 903 [0.1%]), tachypnea (1 of 903 [0.1%]), and ventricular septal defect (1 of 903 [0.1%]).

### Transfusion Volume, Duration, and Infusion Rate

Among the 748 transfusions given based on an Hb threshold, 738 had known volume and duration data (unknown in 10 of 748 transfusions [1.3%]). Most of these were given at a volume of 15 mL/kg (470 [63.7%]), followed by 20 mL/kg (168 [22.8%]). Transfusion volumes were 25 mL/kg in 16 of 738 transfusions (2.2%) (eFigure 2 in Supplement 1). Transfusions were mainly administered over 3 hours (400 [54.2%]) or 4 hours (197 [26.7%]). The infusion rate of most transfusions ranged between 5 to less than 10 mL/kg/h (428 [58.0%]). Data on transfusions given for active bleeding, surgery, or any other primary indication are provided in eFigure 3 in Supplement 1.

### Pretransfusion Hb Level

**Figure 4.**
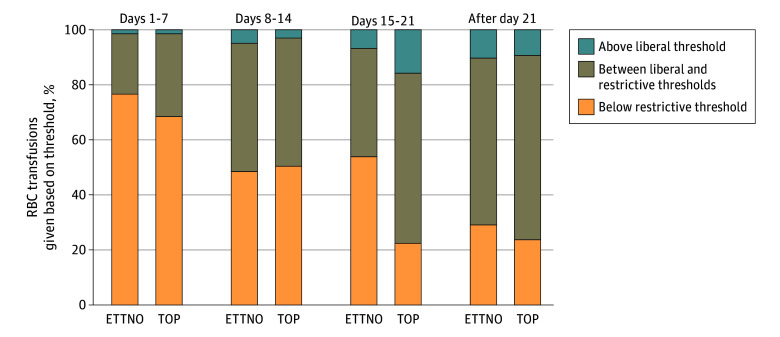
Pretransfusion Hemoglobin (Hb) Values of Transfusions Indicated for Threshold Compared With Previous Trial Thresholds Data were stratified by postnatal age for 729 of 748 transfusions given based on Hb threshold (97.5%). The Hb levels closest prior to transfusion, within 24 hours before and after transfusion are shown. Transfusion thresholds tested in the Effects of Transfusion Thresholds on Neurocognitive Outcomes of Extremely Low-Birth-Weight Infants (ETTNO) and Transfusion of Prematures (TOP) trials are available in eTable 1 in Supplement 1. RBC indicates red blood cell.

Levels of Hb within 24 hours prior to transfusion were available in 729 of the 748 transfusions given based on Hb threshold (97.5%). Compared with ETTNO, 324 transfusions (44.4%) had a pretransfusion Hb level below the evaluated restrictive threshold, 352 (48.3%) were between the evaluated restrictive and liberal thresholds, and 53 (7.3%) were above the evaluated liberal threshold. In contrast, compared with TOP, a lower proportion of transfusions (265 of 729 [36.4%]) had pretransfusion values below the evaluated restrictive threshold, whereas 409 (56.1%) were between the evaluated restrictive and liberal threshold, and 55 (7.5%) were given above the evaluated liberal threshold. The proportion of pretransfusion Hb values below restrictive trial thresholds was higher in early compared with later postnatal ages (Figure 4). There was considerable variation in Hb thresholds, even within similar clinical scenarios, as highlighted by the length of the whiskers in eFigures 4 and 5 in Supplement 1.

### Transfusion Increment

Of the 748 transfusions indicated based on threshold, Hb levels were available both before and after transfusion (within 24 hours before and after transfusion) in 559 (74.7%). The median Hb increment per transfusion was 30 (IQR, 20-39) g/L (eFigure 6 in Supplement 1).

### Transfusion-Associated Adverse Events

Two cases of possible transfusion-associated adverse events were documented among 903 transfusions (0.2%). One record reported culture-proven sepsis following the transfusion. The second reported an increase in oxygen demand after transfusion.

## Discussion

Red blood cell transfusions remain a very common intervention in preterm infants in the NICU, with more than one-third receiving at least 1 transfusion by day 28. This cohort study found substantial differences in RBC transfusion thresholds, volumes, durations, and infusion rates across 22 European countries, which were only partly explained by differences in patient mix. The most common reason for transfusion was a threshold Hb concentration, with a smaller proportion administered for active bleeding or prior to surgery. Most transfusions based on Hb threshold were given for pretransfusion Hb levels above restrictive thresholds as evaluated in the recent ETTNO and TOP RCTs.

The percentage of infants who received at least 1 RBC transfusion in this study was notably lower compared with the ETTNO (702 of 1013 [69.3%]) and TOP (1689 of 1824 [92.6%]) trials.^1,2^ However, these trials included infants with a lower median gestational age at birth than in our study. Variation between countries was only partly explained by patient-mix differences, with some countries appearing to transfuse more and some less than expected based on patient mix. This variability could be explained by various possible factors. For example, transfusion guidelines are expected to vary between countries and centers, given the lack of international consensus. Another explanation for the variability might include different local policies for delayed cord clamping practices and diagnostic blood withdrawal. This association between iatrogenic blood loss and transfusion requirement has been established, and the volume of iatrogenic blood loss may vary between centers depending on the frequency of testing and type of automated analyzers used.^13,14,15^

The primary indication for most of the blood transfusions (82.8%) was Hb threshold. Interestingly, most transfusions based on Hb threshold were given for pretransfusion thresholds above the restrictive thresholds, suggesting that most European countries have not implemented the restrictive thresholds in daily practice. The reasons for this are unclear but may be due to a lack of timely updating of guidelines to implement the evidence from recent trials or to the fact that dissemination of these study results is still in progress. Additionally, secondary clinical reasons other than Hb threshold may have influenced the decision to transfuse above threshold level. Interestingly, the proportion of pretransfusion Hb values above restrictive thresholds tested in the ETTNO and TOP trials was higher in later postnatal weeks compared with the first postnatal week. This finding was contrary to our expectation that clinicians might be more inclined to hold off on a transfusion decision in older infants.

Additionally, a small yet notable proportion of transfusions given based on threshold had pretransfusion Hb levels even above the liberal thresholds tested in recent trials (7.3% and 7.5%).^1,2^ As both trials concluded that liberal thresholds were not superior to restrictive thresholds in terms of mortality or neurodevelopmental impairment at 2 years of corrected age, transfusion for Hb levels above liberal RCT thresholds should be discouraged.

Limited or no evidence is currently available regarding the beneficial effects of transfusion for other indications such as apnea, inadequate weight gain, tachycardia, hypotension, patent ductus arteriosus, and ventricular septal defects.^16,17,18,19,20,21^ However, a decision to transfuse may be justified on an individual basis.

Common volumes for transfusions were 15 to 20 mL/kg, similar to those used in the TOP (15 mL/kg) and ETTNO (20 mL/kg) trials, but with evidence of significant variation.^1,2^ Evidence on optimal volumes for transfusion is scarce. Among the few but all small RCTs assessing infant transfusion volume, 4 trials compared 15 mL/kg with 20 mL/kg,^22,23,24,25^ and 1 compared 10 mL/kg with 20 mL/kg.^26^ These trials found minimal differences in hemodynamic and pulmonary function outcomes between the groups, suggesting that these volumes yield similar physiological responses. The ETTNO and TOP trials did not report information on the duration or infusion rate of transfusions.^1,2^ Transfusion volume influences the risk of transfusion-related circulatory overload and may influence acute lung injury and other adverse events.^27,28,29^ Notably, we observed transfusion volumes of 25 mL/kg in 2.2% of transfusions, which may require extra caution, as the tolerance of these volumes is unknown. Adverse events were reported in only 0.2% of transfusions in our study, corresponding to approximately 2 cases per 100 000 transfusions, and it is not clear if they were causally related to the transfusions. The overall incidence of severe adverse events associated with RBC transfusion in Europe has been estimated at 5 cases per 100 000 RBC units, but this included both adult and pediatric recipients.^30^ Adverse events associated with transfusion in preterm infants are generally poorly defined and are therefore likely to be underrecognized and underreported. Preterm infants are anticipated to be at higher risk for transfusion-associated adverse events considering their vulnerability, supported by existing evidence for potential transfusion-related harm.^31,32,33,34,35^ Clear definitions and accurate registration of these adverse events in preterm infants are urgently needed to allow for a better understanding of the benefit-risk balance of transfusions.

### Strengths and Limitations

Strengths of this study include the large number of participating centers and countries, with which we were able to provide a representative picture of current practice across Europe. The prospective nature of the study allowed us to collect high-quality data on aspects of transfusion practice that are usually not recorded in detail in patient records.

Our study also has several limitations. Center and country results may have been influenced by chance or temporal changes due to the relatively short 6-week study period per center, emphasizing the need for caution when interpreting results on an individual country level. Additionally, the study follow-up duration varied between patients, and not all patients were followed up from birth. However, we used statistical methods that accounted for this. We did not collect information on component specifications of RBC transfusions, as we anticipated that this information was not always available to clinicians.

## Conclusions

This prospective cohort study describing neonatal transfusion practices across Europe found substantial differences in thresholds, volumes, durations, and infusion rates between countries. Research to address these variations and define optimal practices is still needed, including to understand the incomplete uptake of the publications of TOP and ETTNO since 2020. Blood transfusions are biological agents, and the full long-term consequences of transfusions in infants remain unclear.

## Notes

Supplement 1. **eTable 1.** Definitions **eTable 2.** RBC Transfusion Thresholds (Hb in g/L) Tested in the ETTNO and TOP Trials **eTable 3.** Participating Center Characteristics **eFigure 1.** RBC Transfusion Day Prevalence Rates After the First 28 Postnatal Days **eTable 4.** Regression Coefficients for the Estimation of Expected RBC Transfusion Day Prevalence Rates **eFigure 2.** Transfusion Volume, Duration, and Infusion Rate of 738 of 748 Transfusions Given Based on Hb Threshold **eFigure 3.** Transfusion Volume, Duration, and Infusion Rate of 155 Transfusions Given for Bleeding, Surgery, or Any Indication Other Than Hb Threshold **eFigure 4.** Hb Levels Prior to Transfusions Given Based on Hb Threshold **eFigure 5.** Hb Levels Prior to Transfusions Given for Active Bleeding, Surgery, and Other Indications **eFigure 6.** Hb Increment Stratified by Transfusion Volume
eReferences

Supplement 2. **Nonauthor Collaborators.** INSPIRE Study Group Members

Supplement 3.
Data Sharing Statement
