# Error in Supplement 1 and Supplement 2

**DOI:** 10.1001/jamanetworkopen.2024.45861

**Published:** 2024-10-23

In the Original Investigation titled “Red Blood Cell Transfusion in European Neonatal Intensive Care Units, 2022 to 2023,”^1^ published September 19, 2024, there were errors in Supplement 1 and Supplement 2. In eTable 2 of Supplement 1, the acronym in the title given as “ETTNP” should have been “ETTNO.” In Supplement 2, the listings for nonauthor collaborators David Ley and Simone Pratesi were missing. This article was corrected.^1^
