# Supplementary material for: Red Blood Cell Transfusion in European Neonatal Intensive Care Units, 2022 to 2023
Source: JAMA Netw Open. 2024 Sep 19;7(9):e2434077. doi: 10.1001/jamanetworkopen.2024.34077 (PMC11413711; doi:10.1001/jamanetworkopen.2024.34077)
Supplement: Supplement 1. — eTable 1. Definitions eTable 2. RBC Transfusion Thresholds (Hb in g/L) Tested in the ETTNO and TOP Trials eTable 3. Participating Center Characteristics eFigure 1. RBC Transfusion Day Prevalence Rates After the First 28 Postnatal Days eTable 4. Regression Coefficients for the Estimation of Expected RBC Transfusion Day Prevalence Rates eFigure 2. Transfusion Volume, Duration, and Infusion Rate of 738 of 748 Transfusions Given Based on Hb Threshold eFigure 3. Transfusion Volume, Duration, and Infusion Rate of 155 Transfusions Given for Bleeding, Surgery, or Any Indication Other Than Hb Threshold eFigure 4. Hb Levels Prior to Transfusions Given Based on Hb Threshold eFigure 5. Hb Levels Prior to Transfusions Given for Active Bleeding, Surgery, and Other Indications eFigure 6. Hb Increment Stratified by Transfusion Volume eReferences [file jamanetwopen-e2434077-s001.pdf]

Supplementary Online Content

Houben N, Fustolo-Gunnink S, Fijnvandraat K, et al; International Neonatal Transfusion Point Prevalence Study Group. Red blood cell transfusion in European neonatal intensive care units, 2022 to 2023. *JAMA Netw Open*. 2024;7(9):e2434077. doi:10.1001/jamanetworkopen.2024.34077

eTable 1. Definitions

eTable 2. RBC Transfusion Thresholds (Hb in g/L) Tested in the ETTNO and TOP Trials

eTable 3. Participating Center Characteristics

eFigure 1. RBC Transfusion Day Prevalence Rates After the First 28 Postnatal Days

eTable 4. Regression Coefficients for the Estimation of Expected RBC Transfusion Day Prevalence Rates

eFigure 2. Transfusion Volume, Duration, and Infusion Rate of 738 of 748 Transfusions Given Based on Hb Threshold

eFigure 3. Transfusion Volume, Duration, and Infusion Rate of 155 Transfusions Given for Bleeding, Surgery, or Any Indication Other Than Hb Threshold

eFigure 4. Hb Levels Prior to Transfusions Given Based on Hb Threshold

eFigure 5. Hb Levels Prior to Transfusions Given for Active Bleeding, Surgery, and Other Indications

eFigure 6. Hb Increment Stratified by Transfusion Volume

eReferences.

This supplementary material has been provided by the authors to give readers additional information about their work.

© 2024 Houben N et al. *JAMA Network Open*.

eTable 1. Definitions

Event	Definitions
Major congenital anomalies	Chromosomal anomalies, syndromes likely affecting long-term outcome, major malformations requiring surgical correction during newborn period, or cyanotic heart defects
Bleeding disorder	Any genetic or congenital disorders associated with a higher risk of bleeding
Major bleeding	Any of the following bleedings:<br>– Intraventricular haemorrhage (IVH) is defined as IVH Grade 3 (extension of bleeding involving >50% of ventricular area or dilation of ventricle) or IVH Grade 4/IPE (extension of bleeding into surrounding parenchyma) [1]<br>– Intracranial haemorrhage (non-IVH) is defined as a major bleeding if any of the following apply: neurosurgical intervention is required; radiological imaging showing a midline shift; clinical signs and symptoms of an oxygen deficit with significant derangement of laboratory investigations<br>– Pulmonary bleeding is defined as acute fresh blood through the endotracheal tube associated with increased ventilatory requirements or the need for intubation and ventilation<br>– Frank rectal bleeding is defined as macroscopic faecal bleed (not if only occult positive)
Sepsis	Culture proven sepsis
NEC	At least NEC Stage III according to the modified Bell Staging Criteria [2]
High flow respiratory support	High flow (>2L/min) FiO2 ≤0.3 or FiO2>0.3 by non-invasive respiratory support, including continuous positive airway pressure, biphasic intermittent positive airway pressure (synchronized or unsynchronized) and nasal high flow
Invasive mechanical ventilation	By invasive respiratory support for which the infant is intubated, including conventional mechanical ventilation and high frequency oscillation (HFO)
Transfusion associated adverse effect	Any adverse effects that the local investigator deemed potentially associated with the preceeding transfusion.

eTable 2. RBC Transfusion Thresholds (Hb in g/L) Tested in the ETTNO and TOP Trials [3,4]

	ETTNO (Franz et al. JAMA 2020)		TOP (Kirpalani et al. NEJM 2020)	
**Respiratory Support**	**Critical**	**RBC Thresholds**	**Respiratory Support**	**RBC Thresholds**
Room air	No	Day 1-7<br>*Liberal: 117*<br>*Restrictive: 93*<br><br>Day 8-21<br>*Liberal: 103*<br>*Restrictive: 80*<br><br>>Day 21<br>*Liberal: 93*<br>*Restrictive: 70*	No	Day 1-7<br>*Liberal: 120*<br>*Restrictive: 100*<br><br>Day 8-14<br>*Liberal: 110*<br>*Restrictive: 85*<br><br>>Day 15<br>*Liberal: 100*<br>*Restrictive: 70*
Low flow (≤2L/min) FiO_2 >0.21	No	Day 1-7<br>*Liberal: 117*<br>*Restrictive: 93*<br><br>Day 8-21<br>*Liberal: 103*<br>*Restrictive: 80*<br><br>>Day 21<br>*Liberal: 93*<br>*Restrictive: 70*	Yes/No*	Day 1-7<br>*Liberal: 130*<br>*Restrictive: 100*<br><br>Day 8-14<br>*Liberal: 125*<br>*Restrictive: 85*<br><br>>Day 15<br>*Liberal: 110*<br>*Restrictive: 70*
High flow (>2L/min) FiO_2 ≤0.3	Yes/No**	Day 1-7<br>*Liberal: 137*<br>*Restrictive: 93*<br><br>Day 8-21<br>*Liberal: 123*<br>*Restrictive: 80*<br><br>>Day 21<br>*Liberal: 113*<br>*Restrictive: 70*	Yes	Day 1-7<br>*Liberal: 130*<br>*Restrictive: 110*<br><br>Day 8-14<br>*Liberal: 125*<br>*Restrictive: 100*<br><br>>Day 15<br>*Liberal: 110*<br>*Restrictive: 85*
High flow (>2L/min) FiO_2 >0.3	Yes***	Day 1-7<br>*Liberal: 137*<br>*Restrictive: 113*<br><br>Day 8-21<br>*Liberal: 123*<br>*Restrictive: 100*<br><br>>Day 21<br>*Liberal: 113*<br>*Restrictive: 90*	Yes	Day 1-7<br>*Liberal: 130*<br>*Restrictive: 110*<br><br>Day 8-14<br>*Liberal: 125*<br>*Restrictive: 100*<br><br>> Day 15<br>*Liberal: 110*<br>*Restrictive: 85*
Invasive mechanical ventilation	Yes	Day 1-7<br>*Liberal: 137*<br>*Restrictive: 113*<br><br>Day 8-21<br>*Liberal: 123*<br>*Restrictive: 100*<br><br>>Day 21<br>*Liberal: 113*<br>*Restrictive: 90*	Yes	Day 1-7<br>*Liberal: 130*<br>*Restrictive: 110*<br><br>Day 8-14<br>*Liberal: 125*<br>*Restrictive: 100*<br><br>>Day 15<br>*Liberal: 110*<br>*Restrictive: 85*

High flow (>2L/min) FiO2 ≤0.3 or FiO2>0.3 by non-invasive respiratory support, including continuous positive airway pressure (CPAP), biphasic intermittent positive airway pressure (synchronized or unsynchronized) and nasal high flow. Mechanical

ventilation by invasive respiratory support for which the infant is intubated, including conventional mechanical ventilation and high frequency oscillation. *Categorized as both ‘respiratory support’ and ‘no respiratory support’ per TOP definition, respiratory support if flow ≥1 liter per min. **Categorized as both ‘critical’ and ‘noncritical’ per ETTNO definition, critical if CPAP with FiO2 >0.25 for >12 hours per 24 hours. Highest liberal and lowest restrictive transfusion threshold values were selected if scenarios could be categorized as both. ***Categorized as ‘critical’ based on expert opinion, considering that the majority of infants on high flow (>2L/min) FiO2 >0.3 are criticially ill and require respiratory support for more than 12 hours per 24 hours. ETTNO thresholds were converted using the formula: Hb (g/L) = Hematocrit (Hct) (%) / 0.3.

eTable 3. Participating Center Characteristics

Country	Participating centers (n (% of total)	Centers with academic status (n (%))	Centers that perform NEC surgery (n (%))	Large centers* (n (%))
Austria	2 (3%)	2/2 (100)	2/2 (100)	1/2 (50)
Belgium	1 (2%)	1/1 (100)	1/1 (100)	1/1 (100)
Bosnia and Herzegovina	1 (2%)	1/1 (100)	1/1 (100)	1/1 (100)
Croatia	2 (3%)	2/2 (100)	1/2 (50)	1/2 (50)
Czech Republic	2 (3%)	2/2 (100)	1/2 (50)	2/2 (100)
Denmark	1 (2%)	1/1 (100)	1/1 (100)	1/1 (100)
France	6 (9%)	3/6 (50)	4/6 (67)	3/6 (50)
Germany	4 (6%)	4/4 (100)	3/4 (75)	2/4 (50)
Hungary	3 (5%)	3/3 (100)	1/3 (33)	3/3 (100)
Ireland	2 (3%)	2/2 (100)	0/2 (0)	2/2 (100)
Italy	8 (13%)	8/8 (100)	7/8 (88)	1/8 (13)
Netherlands	3 (5%)	3/3 (100)	2/3 (67)	3/3 (100)
Norway	2 (3%)	2/2 (100)	1/2 (50)	0/2 (0)
Poland	5 (8%)	4/5 (80)	3/5 (60)	1/5 (20)
Portugal	2 (2%)	2/2 (100)	2/2 (100)	0/2 (0)
Romania	3 (5%)	2/3 (67)	0/3 (0)	3/3 (100)
Slovakia	2 (3%)	2/2 (100)	1/2 (50)	0/2 (0)
Slovenia	2 (3%)	2/2 (100)	1/2 (50)	1/2 (50)
Spain	6 (9%)	6/6 (100)	5/6 (83)	3/6 (50)
Sweden	3 (5%)	3/3 (100)	2/3 (67)	2/3 (67)
Switzerland	2 (3%)	2/2 (100)	2/2 (100)	1/2 (50)
United Kingdom	2 (3%)	2/2 (100)	1/2 (50)	1/2 (50)
Overall	64/64 (100%)	59/64 (92%)	42/64 (66%)	33/64 (52%)

*Large if center cares for more than 100 preterm infants born below 32 weeks gestation annually.

eFigure 1. RBC Transfusion Day Prevalence Rates After the First 28 Postnatal Days

**Country**	**Observed RBC transfusion days** Per 100 admission days (95% CI)	**Expected RBC transfusion days*** Per 100 admission days
Belgium	0.5 (0.1-3.3)	1.2
Germany	0.8 (0.4-1.5)	2.4
Netherlands	1.0 (0.4-2.7)	2.6
Spain	1.2 (0.3-4.8)	2.4
Czech Republic	1.3 (0.3-5.8)	2.0
Slovakia	1.4 (0.3-6.0)	2.2
Portugal	1.4 (0.5-4.4)	1.5
Denmark	1.4 (0.6-3.4)	3.4
France	1.5 (0.7-3.1)	2.8
Ireland	2.0 (1.0-3.8)	2.4
Slovenia	2.0 (0.4-9.5)	2.0
Croatia	2.0 (0.9-4.5)	2.7
Switzerland	2.5 (1.4-4.5)	3.1
Italy	2.7 (1.5-4.8)	2.6
Norway	2.7 (0.4-20.7)	4.2
Austria	2.8 (1.9-4.2)	2.1
Poland	3.8 (2.3-6.5)	2.9
Romania	4.0 (2.2-7.3)	2.6
Sweden	4.1 (2.3-7.2)	2.7
United Kingdom	4.1 (0.6-27.6)	6.4
Bosnia and Herzegovina	5.1 (2.9-9.0)	2.3
Hungary	8.7 (5.7-13.3)	4.6
**Overall**	**2.0 (1.6-2.6)**	

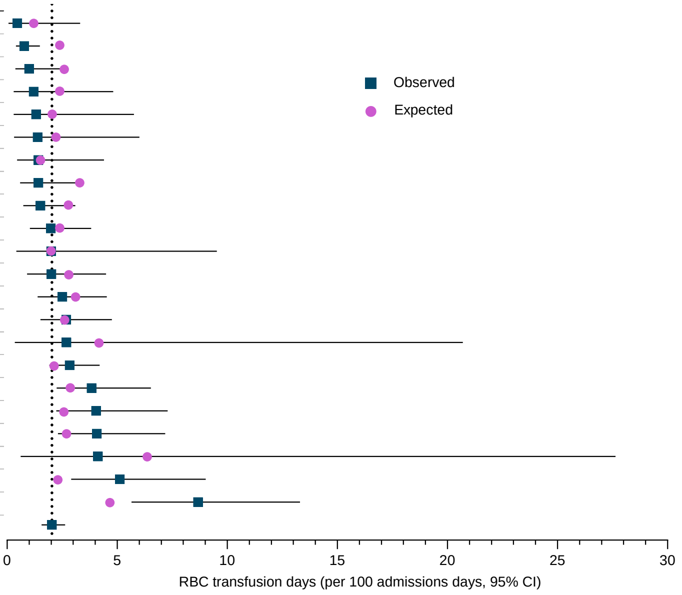

Observed prevalence rates were calculated using random effects Poisson models to pool RBC transfusion day prevalence rates from the individual centers into country subgroup estimates and subsequently to derive the overall estimate. Expected RBC transfusion day prevalence rates as predicted based on patient-mix using a logistic regression model which included the following variables: sex, multiple gestation, gestational age at birth, birth weight, postnatal day, major congenital anomalies, bleeding disorders, major bleeding, NEC, sepsis, mechanical ventilation, surgery.

eTable 4. Regression Coefficients for the Estimation of Expected RBC Transfusion Day Prevalence Rates

	Regression 1. During postnatal day 1-28		Regression 2. After postnatal day 28	
Variable	Coefficient (β)	95% CI (based on asymptotic normality)	Coefficient (β)	95% CI (based on asymptotic normality)
(Intercept)	-0.29	-2.27, 1.68	-2.77	-5.32, -0.21
Sex*	-0.15	-0.37, 0.08	-0.27	-0.51, -0.02
Bleeding*	0.83	0.48, 1.18	0.32	-0.33, 0.98
Status post bleeding*	-0.21	-0.77, 0.34	-0.06	-0.62, 0.49
Postnatal age, in days	-0.01	-0.02, 0.00	-0.01	-0.02, 0.00
Surgery*	0.75	0.26, 1.24	0.76	0.29, 1.24
Status post surgery*	0.94	0.51, 1.34	0.24	-0.10, 0.58
Ventilation*	1.41	1.09, 1.74	1.43	1.09, 1.78
Status post ventilation*	0.75	0.42, 1.08	0.46	0.12, 0.80
Sepsis*	0.82	0.50, 1.13	1.35	0.83, 1.86
Status post sepsis*	0.29	-0.16, 0.73	0.49	0.27, 0.75
NEC*	0.34	-0.31, 0.98	1.00	0.52, 1.49
Status post NEC*	-1.94	-3.11, -0.77	0.16	-0.57, 0.88
Gestational age at birth, in days	-0.01	-0.02, 0.00	0.00	-0.02, 0.01
Multiparity*	-0.20	-0.46, 0.05	-0.22	-0.51, 0.06
Congenital malformations*	0.09	-0.47, 0.66	-0.22	-0.77, 0.33
Bleeding disorder*	1.33	-0.17, 2.83	1.58	0.84, 2.32
Birth weight, in grams	0.00	0.00, 0.00	0.00	0.00, 0.00

*Variables: sex (0=male, 1=female), bleeding (0=no, 1=yes), status post bleeding (0=no, 1=yes), surgery (0=no, 1=yes), status post surgery (0=no, 1=yes), ventilation (0=no, 1=yes), status post ventilation (0=no, 1=yes), sepsis (0=no, 1=yes), status post sepsis (0=no, 1=yes), NEC (0=no, 1=yes), status post NEC (0=no, 1=yes), multiparity (0=no, 1=yes), congenital malformations (0=no, 1=yes), bleeding disorder (0=no, 1=yes). Definitions are available in eTable 1 in the Supplement.

eFigure 2. Transfusion Volume, Duration, and Infusion Rate of 738 of 748 Transfusions Given Based on Hb Threshold

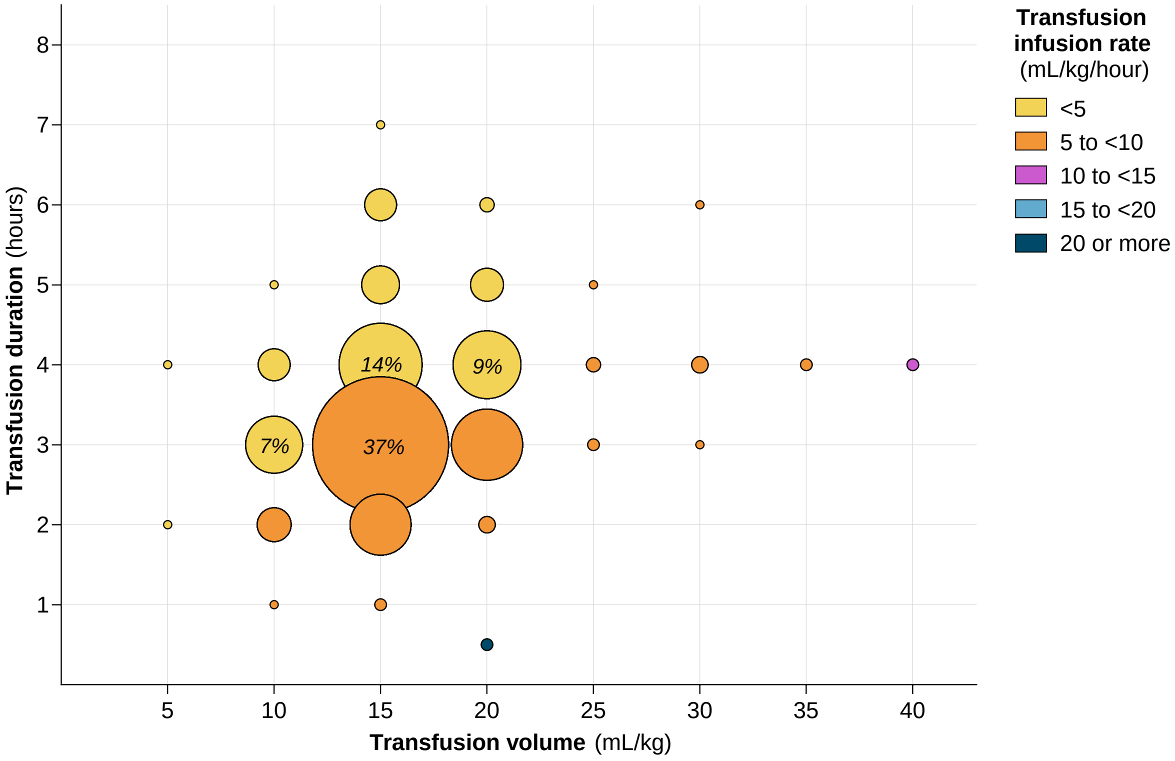

Volume and/or duration unknown in 10 transfusions. Transfusion volumes were 5 mL/kg or less in 0.3% (2/738), 10 mL/kg in 11.1% (82/738), 15 mL/kg in 63.7% (470/738), 20 mL/kg in 22.8% (168/738), 25 mL/kg in 0.8% (6/738), 30 mL/kg in 0.8% (6/738), 35 mL/kg in 0.3% (2/738), and 40 mL/kg in 0.3% (2/738). Transfusion durations were 30 minutes or less in 0.3% (2/738), 1 hour in 0.4% (3/738), 2 hours in 10.4% (77/738), 3 hours in 54.2% (400/738), 4 hours in 26.7% (197/738), 5 hours in 5.3% (39/738), 6 hours in 2.6% (19/738) and 7 hours in 0.1% (1/738) of transfusions.

eFigure 3. Transfusion Volume, Duration, and Infusion Rate of 155 Transfusions Given for Bleeding, Surgery, or Any Indication Other Than Hb Threshold

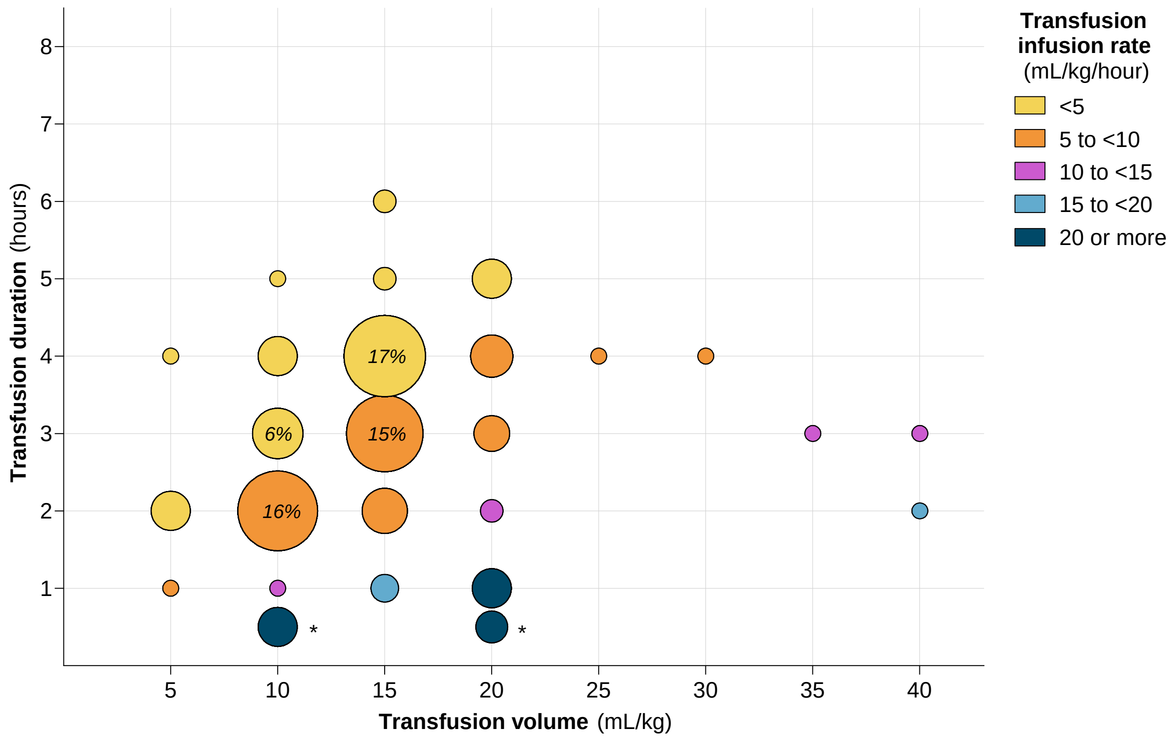

*Transfusion duration 30 minutes or less.

eFigure 4. Hb Levels Prior to Transfusions Given Based on Hb Threshold

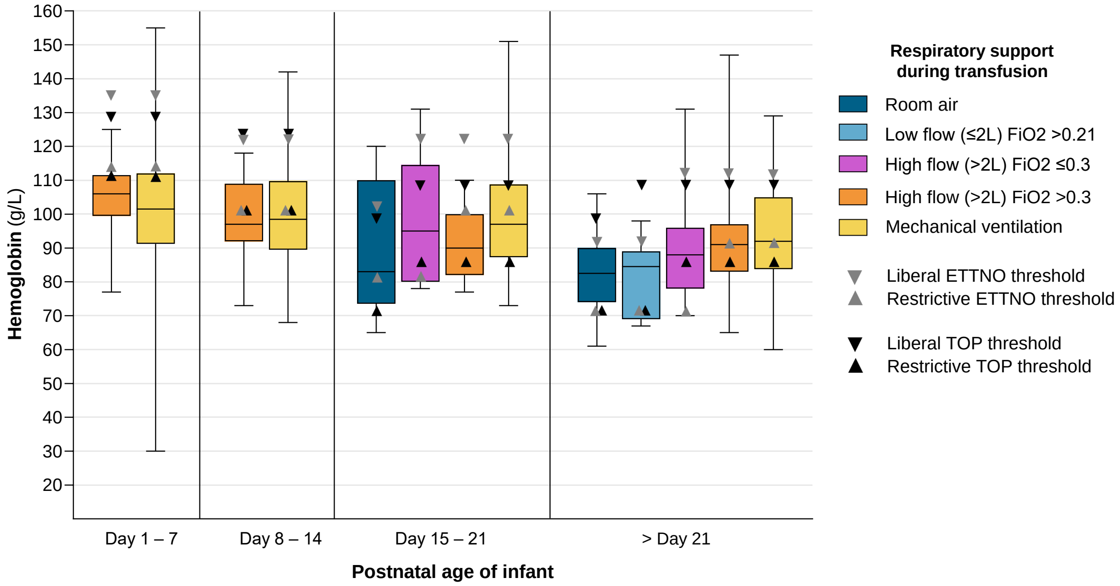

Hb levels prior to transfusion were available in 729 out of 748 transfusions given based on threshold (97.5%). High flow (>2L/min) FiO2 ≤0.3 or FiO2>0.3 by non-invasive respiratory support, including continuous positive airway pressure, biphasic intermittent positive airway pressure (synchronized or unsynchronized) and nasal high flow. Mechanical ventilation by invasive respiratory support for which the infant is intubated, including conventional mechanical ventilation and high frequency oscillation. Boxplots with fewer than 5 transfusions were omitted from the plot (n=13 transfusions). The middle line of the box denotes the median, the ends of the box denote the 25th to 75th percentile, the whiskers denote the minimum to maximum range. ETTNO and TOP thresholds are available in Supplement 3. eTable 2. If respiratory support scenarios in this study could be categorized as both 'critical' or 'noncritical' according to the ETTNO trial or as both 'respiratory support' and 'no respiratory support' according to TOP trial, the highest liberal and lowest restrictive value were selected.

eFigure 5. Hb Levels Prior to Transfusions Given for Active Bleeding, Surgery, and Other Indications

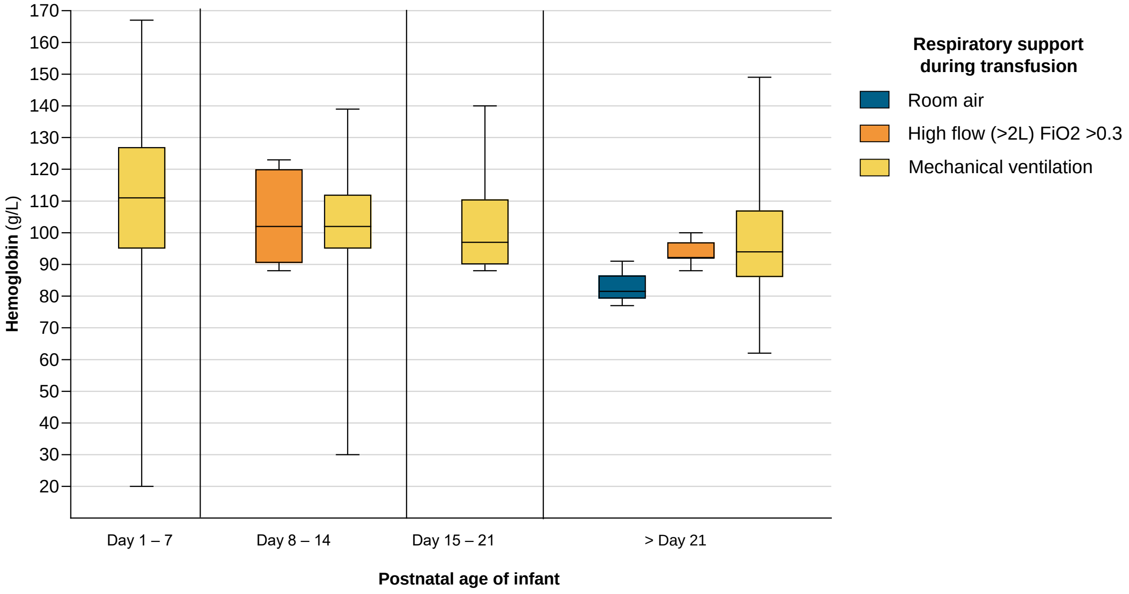

Hb levels prior to transfusion were available in 147 out of 155 transfusions given for active bleeding, surgery, and other indications (92.9%). High flow (>2L/min) FiO2 ≤0.3 or FiO2>0.3 by non-invasive respiratory support, including continuous positive airway pressure (CPAP), biphasic intermittent positive airway pressure (synchronized or unsynchronized) and nasal high flow. Mechanical ventilation by invasive respiratory support for which the infant is intubated, including conventional mechanical ventilation and high frequency oscillation. Boxplots with fewer than 5 transfusions were omitted from the plot (n=11 transfusions). The middle line of the box denotes the median, the ends of the box denote the 25th to 75th percentile, the whiskers denote the minimum to maximum range.

eFigure 6. Hb Increment Stratified by Transfusion Volume

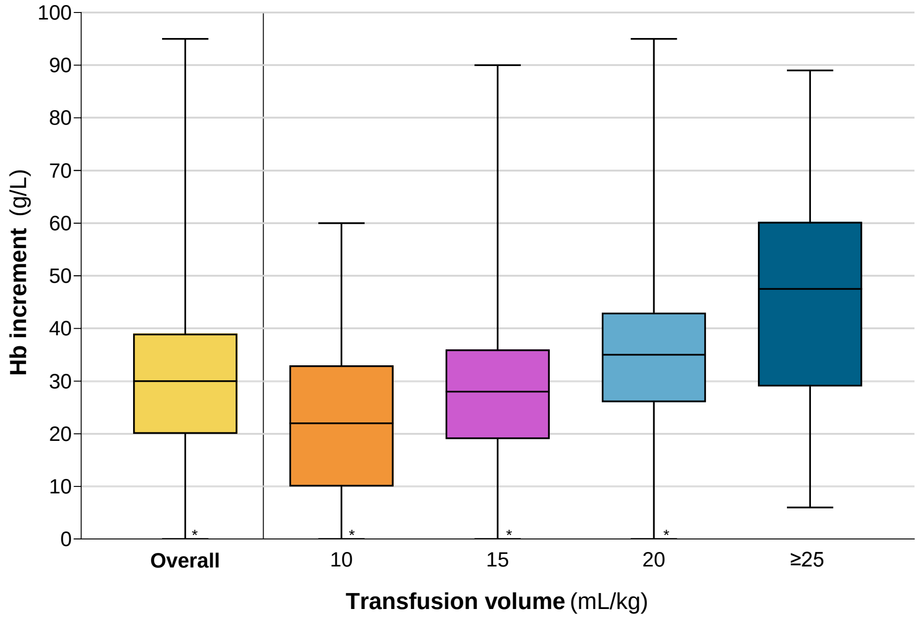

In 559 of 748 (74.7%) transfusions given based on threshold, both pre-transfusion Hb and post-transfusion Hb levels were available. Boxplots with fewer than 5 transfusions were omitted from the plot (n=1 transfusion). *Negative Hb increment in 2 transfusions at 10ml/kg, 7 transfusions at 15 ml/kg and 1 transfusion at 20ml/kg plotted outside axis limits. The middle line of the box denotes the median, the ends of the box denote the 25th to 75th percentile, the whiskers denote the minimum to maximum range.

eReferences

1. Papile LA, Burstein J, Burstein R, Koffler H. Incidence and evolution of subependymal and intraventricular hemorrhage: a study of infants with birth weights less than 1,500 gm. *J Pediatr*. Apr 1978;92(4):529-34. doi:10.1016/s0022-3476(78)80282-0

2. Bell MJ. Neonatal necrotizing enterocolitis. *N Engl J Med*. Feb 2 1978;298(5):281-2.

3. Franz AR, Engel C, Bassler D, et al. Effects of Liberal vs Restrictive Transfusion Thresholds on Survival and Neurocognitive Outcomes in Extremely Low-Birth-Weight Infants: The ETTNO Randomized Clinical Trial. *Jama*. 2020;324(6):560-570. doi:10.1001/jama.2020.10690

4. Kirpalani H, Bell EF, Hintz SR, et al. Higher or Lower Hemoglobin Transfusion Thresholds for Preterm Infants. *New England Journal of Medicine*. 2020;383(27):2639-2651. doi:10.1056/NEJMoa2020248
