# Supplementary material for: Red Blood Cell Transfusion in European Neonatal Intensive Care Units, 2022 to 2023
Source: JAMA Netw Open. 2024 Sep 19;7(9):e2434077. doi: 10.1001/jamanetworkopen.2024.34077 (PMC11413711; doi:10.1001/jamanetworkopen.2024.34077)
Supplement: Supplement 2. — Nonauthor Collaborators. INSPIRE Study Group Members [file jamanetwopen-e2434077-s002.pdf]

Supplemental Online Content: Nonauthor Collaborators
*First name, last name, and suffix (if applicable) are required and will appear in PubMed.

***Group Name(s): INSPIRE Study Group**

| ***First Name and Middle Initial(s)** | ***Last Name** | ***Suffix (eg, Jr, III)** | Academic Degrees | Institution | Location (city, state/province, country) | Role or Contribution, eg, chair, principal investigator | Group (if more than 1 Group listed in the byline) and/or Subgroup (eg, Steering Committee) |
|---|---|---|---|---|---|---|---|
| Miguel | Alsina-Casanova | | | Clínic Barcelona Hospital Universitari | Barcelona, Spain | Local investigator | |
| Ola | Andersson | | | Lund University and Skane University Hospital | Lund, Sweden | Local investigator | |
| Rosa Patricia | Arias-Llorente | | | Central University Hospital of Asturias | Oviedo, Spain | Local investigator | |
| Adeline | Berenger | | | CHU Limoges | Limoges, France | Local investigator | |
| Edyta | Bielska | | | Department of Neonatology and Neonatal Intensive Care Medical University of Warsaw | Warsaw, Poland | Local investigator | |
| Marioara | Boia | | | Spitalul Clinic de Urgenta Pentru Coppii Louis Turcanu | Timisoara, Romania | Local investigator | |
| André | Birkenmaier | | | University of Zurich and Children's Hospital St. Gallen | St. Gallen, Switzerland | Local investigator | |
| Jakub | Biros | | | Faculty Hospital Nove Zamky | Nove Zamky, Slovakia | Local investigator | |
| Anne Laure | Blanquart | | | CHU Limoges | Limoges, France | Local investigator | |
| Tiziana | Boggini | | | Fondazione IRCCS Policlinico San Matteo | Pavia, Italy | Local investigator | |
| Pascal | Boileau | | | CHI Poissy - Saint-Germain-en-Laye | Poissy, France | Local investigator | |
| Renata | Bokiniec | | | Department of Neonatology and Neonatal Intensive Care Medical University of Warsaw | Warsaw, Poland | Local investigator | |
| Ilia | Bresesti | | | Filippo Del Ponte Hospital, University of Insubria | Varese, Italy | Local investigator | |
| Katherine | Broad | | | St Michael's Hospital, University Hospitals Bristol and Weston | Bristol, United Kingdom | Local investigator | |

Supplemental Online Content: Nonauthor Collaborators
*First name, last name, and suffix (if applicable) are required and will appear in PubMed.

| ***First Name and Middle Initial(s)** | ***Last Name** | ***Suffix (eg, Jr, III)** | Academic Degrees | Institution | Location (city, state/province, country) | Role or Contribution, eg, chair, principal investigator | Group (if more than 1 Group listed in the byline) and/or Subgroup (eg, Steering Committee) |
|---|---|---|---|---|---|---|---|
| Giacomo | Cavallaro | | | Neonatal Intensive Care Unit, Fondazione IRCCS Ca' Granda Ospedale Maggiore Policlinico | Milano, Italy | Local investigator | |
| Jennifer | Chauvel | | | CH de Saint Brieuc | Saint-Brieuc, France | Local investigator | |
| Borbála | Cseszneki | | | Semmelweis University | Budapest, Hungary | Local investigator | |
| Carlo | Dani | | | Azienda Ospedaliero Universitaria Careggi | Florence, Italy | Local investigator | |
| Klaudia | Demová | | | Faculty Hospital Nove Zamky | Nove Zamky, Slovakia | Local investigator | |
| Diana | Dornis | | | Universitätsklinikum Leipzig | Leizpig, Germany | Local investigator | |
| Marie-Pierre | Duban | | | CH de Saint Brieuc | Saint-Brieuc, France | Local investigator | |
| Karolina | Dziadkowiec-Motyl | | | Górnośląskie Centrum Zdrowia Dziecka, Medical Faculty Silesian Medical University | Katowice, Poland | Local investigator | |
| Nika | Erzen | | | University Medical Center Ljubljana | Ljubljana, Slovenia | Local investigator | |
| Eszter | Fanczal | | | Semmelweis University | Budapest, Hungary | Local investigator | |
| Sara | Fernández-Castiñeira | | | Central University Hospital of Asturias | Oviedo, Spain | Local investigator | |
| Libusa | Galuschka | | | Klinikum Lüneburg | Lüneburg, Germany | Local investigator | |
| Ellen | Gandaputra | | | Klinikum Lüneburg | Lüneburg, Germany | Local investigator | |
| Fermín | García-Muñoz Rodrigo | | | CHU Insular-Materno Infantil | Las Palmas, Spain | Local investigator | |
| Corinna | Gebauer | | | Universitätsklinikum Leipzig | Leizpig, Germany | Local investigator | |
| Hélène | Grimault | | | CH Bretagne-Atlantique | Vannes, France | Local investigator | |
| Kristina | Grund | | | Department of Neonatology, University Children's Hospital Regensburg (KUNO), Hospital St. Hedwig of the Order of St. John, University of Regensburg | Regensburg, Germany | Local investigator | |
| Melanie | Gsöllpointner | | | Kepler University Hospital | Linz, Austria | Local investigator | |
| Silvia | Gualdi | | | IRCCS AOU S. Orsola | Bologna, Italy | Local investigator | |

Supplemental Online Content: Nonauthor Collaborators
*First name, last name, and suffix (if applicable) are required and will appear in PubMed.

| ***First Name and Middle Initial(s)** | ***Last Name** | ***Suffix (eg, Jr, III)** | Academic Degrees | Institution | Location (city, state/province, country) | Role or Contribution, eg, chair, principal investigator | Group (if more than 1 Group listed in the byline) and/or Subgroup (eg, Steering Committee) |
|---|---|---|---|---|---|---|---|
| Brunetta | Guaragni | | | Spedali Civili di Brescia | Brescia, Italy | Local investigator | |
| Markus | Hahn | | | University of Zurich and Children's Hospital St. Gallen | St. Gallen, Switzerland | Local investigator | |
| Nadja | Haiden | | | Kepler University Hospital | Linz, Austria | Local investigator | |
| Monica | Hasmasanu | | | University of Medicine and Pharmacy Iuliu Hatieganu | Cluj-Napoca, Romania | Local investigator | |
| Daniela | Iacob | | | Timisoara County Emergency Clinical Hospital | Timisoara, Romania | Local investigator | |
| Mihaela | Ivanici | | | Spitalul Clinic de Urgenta Pentru Coppii Louis Turcanu | Timisoara, Romania | Local investigator | |
| Raphaela | Jernej | | | Medical University Vienna | Vienna, Austria | Local investigator | |
| Tomáš | Juren | | | University Hospital Brno | Brno, Czech Republic | Local investigator | |
| Karolina | Karcz | | | Department of Neonatology Wroclaw Medical University | Wrocław, Poland | Local investigator | |
| Lilijana | Kornhauser | | | Maternity Hospital Ljubljana | Ljubljana, Slovenia | Local investigator | |
| Barbara | Królak-Olejnik | | | Department of Neonatology Wroclaw Medical University | Wrocław, Poland | Local investigator | |
| Lena | Legnevall | | | Karolinska Institute | Stockholm, Sweden | Local investigator | |
| Verena | Lehnerer | | | Department of Neonatology, University Children's Hospital Regensburg (KUNO), Hospital St. Hedwig of the Order of St. John, University of Regensburg | Regensburg, Germany | Local investigator | |
| Emmanuelle | Levine | | | CHU de Rennes | Rennes, France | Local investigator | |
| David | Ley | | | Lund University and Skane University Hospital | Lund, Sweden | Local investigator | |
| María Del Carmen | López Castillo | | | Hospital Regional Universitario de Málaga | Málaga, Spain | Local investigator | |
| Mariella | Magarotto | | | Azienda Ospedale Università Padova | Padova, Italy | Local investigator | |
| Silvia | Martini | | | IRCCS AOU S. Orsola | Bologna, Italy | Local investigator | |

*First name, last name, and suffix (if applicable) are required and will appear in PubMed.

| ***First Name and Middle Initial(s)** | ***Last Name** | ***Suffix (eg, Jr, III)** | Academic Degrees | Institution | Location (city, state/province, country) | Role or Contribution, eg, chair, principal investigator | Group (if more than 1 Group listed in the byline) and/or Subgroup (eg, Steering Committee) |
|---|---|---|---|---|---|---|---|
| Iwona | Maruniak-Chudek | | | Górnośląskie Centrum Zdrowia Dziecka, Medical Faculty Silesian Medical University | Katowice, Poland | Local investigator | |
| Rita | Moita | | | CHU de São João | Porto, Portugal | Local investigator | |
| Anjola | Mosuro | | | Great Western Hospitals | Wiltshire, United Kingdom | Local investigator | |
| Agnieszka | Nowicka | | | Centrum Medyczne Ujastek, Kraków | Kraków, Poland | Local investigator | |
| Daniel | O'Reilly | | | Rotunda Hospital | Dublin, Ireland | Local investigator | |
| Manuela | Pantea | | | Timisoara County Emergency Clinical Hospital | Timisoara, Romania | Local investigator | |
| Alejandro | Pérez-Muñuzuri | | | Hospital Clínico Universitario de Santiago | Santiago de Compostela, Spain | Local investigator | |
| Tina | Perme | | | Maternity Hospital Ljubljana | Ljubljana, Slovenia | Local investigator | |
| Laura | Picciau | | | Spedali Civili di Brescia | Brescia, Italy | Local investigator | |
| Simone | Pratesi | | | Azienda Ospedaliero Universitaria Careggi | Florence, Italy | Local investigator | |
| Sandra | Prins | | | Emma Children’s Hospital Amsterdam University Medical Center, Department of Neonatology | Amsterdam, The Netherlands | Local investigator | |
| Maurizio | Radicioni | | | Azienda Opsedaliera di Perugia | Perugia, Italy | Local investigator | |
| Genny | Raffaeli | | | Neonatal Intensive Care Unit, Fondazione IRCCS Ca' Granda Ospedale Maggiore Policlinico | Milano, Italy | Local investigator | |
| Reyes | Roldan-López | | | Hospital Regional Universitario de Málaga | Málaga, Spain | Local investigator | |
| Jean-Michel | Roué | | | CHRU Brest | Brest, France | Local investigator | |
| Beata | Rzepecka Węglarz | | | Centrum Medyczne Ujastek, Kraków | Kraków, Poland | Local investigator | |

Supplemental Online Content: Nonauthor Collaborators
*First name, last name, and suffix (if applicable) are required and will appear in PubMed.

| ***First Name and Middle Initial(s)** | ***Last Name** | ***Suffix (eg, Jr, III)** | Academic Degrees | Institution | Location (city, state/province, country) | Role or Contribution, eg, chair, principal investigator | Group (if more than 1 Group listed in the byline) and/or Subgroup (eg, Steering Committee) |
|---|---|---|---|---|---|---|---|
| Greta | Sibrecht | | | II Department of Neonatology, Poznan University of Medical Sciences | Poznan, Poland | Local investigator | |
| Pauline | Snijder | | | Erasmus MC | Rotterdam, The Netherlands | Local investigator | |
| Mirta | Starčević | | | University Hospital Centre Zagreb | Zagreb, Croatia | Local investigator | |
| Emese | Szántó | | | Semmelweis University | Budapest, Hungary | Local investigator | |
| Liliana | Teixeira | | | Centro Materno-Infantil do Norte - Unidade Local de Saúde de Santo António | Porto, Portugal | Local investigator | |
| Laura | Torrejon | | | La Fe University Hospital | Valencia, Spain | Local investigator | |
| Lourdes | Urquía Martí | | | CHU Insular-Materno Infantil | Las Palmas, Spain | Local investigator | |
| Laurien | Vanbuggenhout | | | UZ Leuven | Leuven, Belgium | Local investigator | |
| Lorenzo | Zanetto | | | Azienda Ospedale Università Padova | Padova, Italy | Local investigator | |
