# Supplementary material for: Red Blood Cell Transfusion in European Neonatal Intensive Care Units, 2022 to 2023
Source: JAMA Netw Open. 2024 Sep 19;7(9):e2434077. doi: 10.1001/jamanetworkopen.2024.34077 (PMC11413711; doi:10.1001/jamanetworkopen.2024.34077)
Supplement: Supplement 3. — Data Sharing Statement [file jamanetwopen-e2434077-s003.pdf]

# Data Sharing Statement

Houben. Red Blood Cell Transfusion in European Neonatal Intensive Care Units, 2022 to 2023. *JAMA Netw Open*. Published September 19, 2024.
doi:10.1001/jamanetworkopen.2024.34077

**Data**
**Data available:** No

**Additional Information**
**Explanation for why data not available:** No consent was obtained for data sharing. External applications for data access will therefore only be taken into consideration after the prior written consent of all participating centers.
